# Influence of furrow irrigation regime on the yield and water consumption indicators of winter wheat based on a multi-level fuzzy comprehensive evaluation

**DOI:** 10.1515/biol-2022-0059

**Published:** 2022-09-08

**Authors:** Xiao-Hang Li, Kun Sheng, Ying-Hong Wang, Yan-Qi Dong, Zhi-Kai Jiang, Jing-Sheng Sun

**Affiliations:** Farmland Irrigation Research Institute, Chinese Academy of Agricultural Sciences/Key Laboratory of Crop Water Use and Regulation, Ministry of Agriculture, Xinxiang 453000, Henan, P. R. China; Institute of Wheat Research, Xinxiang Academy of Agricultural Sciences, Xinxiang 453000, Henan, P. R. China

**Keywords:** winter wheat, irrigation regime, multi-level, crop water productivity, entropy weight method, model fusion

## Abstract

Irrigation regimes should be chosen to maximize crop yield and water use efficiency. To realize high yield and efficient water use with the appropriate furrow irrigation regime, the effects of two regimes (alternate furrow irrigation and conventional furrow irrigation) and three lower soil moisture limits (60, 70, and 80%) were studied on winter wheat yield and water consumption using a multi-level fuzzy comprehensive evaluation method. The results show that under the two regimes, alternate furrow irrigation and conventional furrow irrigation, when the lower limit of the soil moisture is 70%, the harvest index (0.45 and 0.39, respectively) and crop water productivity of winter wheat (1.86 and 1.90 kg m^−3^, respectively) are highest. The comprehensive fuzzy evaluation model considers multiple measures, including yield, harvest indices, irrigation volume, total water consumption, and crop water productivity – the index values are highest at the 70% condition, which are 0.3468 and 0.3432, respectively. Therefore, it can be concluded that a moderate water deficit is conducive to saving water resources and improving water use efficiency. In conclusion, a multi-level and multi-factor indices system of furrow irrigation regime was constructed based on ensuring winter wheat production. Conventional furrow irrigation is recommended in areas with sufficient irrigation water, while alternating furrow irrigation, which can reduce the total amount of irrigation required, is suitable for areas with water shortages.

## 1 Introduction

The North China Plain, one of the main wheat-producing areas in China, provides about 50% of the national output of wheat each year [1]. However, the period of relatively low precipitation in this area coincides with the wheat growing period, such that the wheat development cannot rely solely on precipitation. Therefore, it is necessary to supplement irrigation to achieve a stable and high yield. At present, efficient water-saving technology and methods for improving the water use efficiency of wheat are usually used to alleviate the contradiction between the supply and demand of water resources [2]. Previous studies have shown that the speed of water flow in the irrigation of wheat planting in traditional border fields will be affected by the length of the border field and ground flatness, resulting in problems such as time-consuming irrigation period and waste of irrigation water [3].

Planting crops by mechanical furrow and ridge in a field can improve the efficiency of irrigation and facilitate the collection of precipitation in associated ditches. Natural precipitation is closer to the root of crops, the water content of field soil increases, and the external water supply is enhanced, improving water use efficiency while increasing crop yield [4]. When planting crops on a ridge, corrugation irrigation is adopted. The water in an irrigation ditch seeps to the ridge under the action of water potential differences [5]. Therefore, changing the traditional planting mode to ridge planting can effectively coordinate the relationship between wheat groups and individuals and promote wheat individuals to be more robust, with moderate group size and significant edge row advantages (e.g., spike number, grain numbers per spike, and 1,000-grain weight). Under this planting mode, wheat production can increase by about 10% [6]. Wang et al. showed that ridge furrow irrigation could reduce water consumption by about 30% compared with traditional border irrigation [7]. The research of Ma et al. and Moayeri et al. found that the total water consumption of ridge farming was significantly reduced, and the water use efficiency was improved during the growth period of the cultivated crops [8,9].

The combination of alternate irrigation and furrow irrigation is called alternate furrow irrigation [10,11,12,13,14]. The non-irrigated ditch in the process of alternate furrow irrigation produces drought stress on crops and promotes their root growth to improve water use efficiency [15,16,17,18]. The plant has specific sensing and signal transmission systems, which can quickly recognize the change in soil water content and transmit a signal to the guard cells located in the leaves to reduce the stomatal conductance and then reduce the transpiration of plants [19,20,21,22,23,24]. The changing trend of stomatal conductance is inconsistent between transpiration and water consumption, the former being linear while the latter being gradually saturated. If the stomatal conductance is properly reduced, the transpiration water loss can be significantly reduced, and it has little effect on photosynthesis [25,26,27,28,29,30]. The disadvantages of conventional furrow irrigation technology can be greatly improved through controlled alternate furrow irrigation, which can significantly reduce within tree evaporation, reduce crop transpiration water loss, and improve crop water use efficiency [31,32]. The research shows that the alternate furrow irrigation can significantly reduce the water loss via leaf transpiration but will not significantly reduce the photosynthetic rate [33,34,35].

Different furrow irrigation regimes have different effects on wheat growth and development, grain yield, and water use efficiency. Through the analysis of these important measures, we can select the appropriate furrow irrigation regime to maximize economic and ecological benefits of water. At present, the comprehensive analysis method is generally used, which uses correlation or causality comparisons between a single index and multi-indices for analysis [36,37]. The comprehensive analysis method is influenced by researchers’ subjective thoughts, which results in the uncertainty of the evaluation results [38]. Therefore, it is of significance to establish a scientific evaluation model with the help of mathematical principles to clarify the best furrow irrigation scheme. Because of its advantages in dealing with multiple indicators, fuzzy comprehensive evaluation has achieved robust results in climate change simulation research, agricultural machinery structure optimization, and precision gear manufacturing [39,40,41,42,43,44]. This study aims to establish a scientific evaluation of the three factors of wheat yield and water use efficiency through the multi-level fuzzy evaluation method. According to this evaluation, a more efficient furrow irrigation regime for North China is suggested, and a theoretical basis for realizing the scientific irrigation, high yield, and quality of wheat is provided.

## 2 Materials and methods

### 2.1 Test site

The experiment was conducted in the Huixian experimental base (36°9′ N, 113°7′ E) of Xinxiang Academy of Agricultural Sciences from 2016 to 2017. Before wheat sowing, the soil layer of 0–20 cm contained 14.14 g kg^−1^ organic matter, 1.08 g kg^−1^ total nitrogen, 11.39 mg kg^−1^ available phosphorus, 111.2 mg kg^−1^ available potassium, pH = 8.16, the bulk density of the 0–200 cm soil layer was 1.45 g cm^3^, and the field water holding capacity was 32% (saturated moisture content).

### 2.2 Experimental design

**Figure 1:**
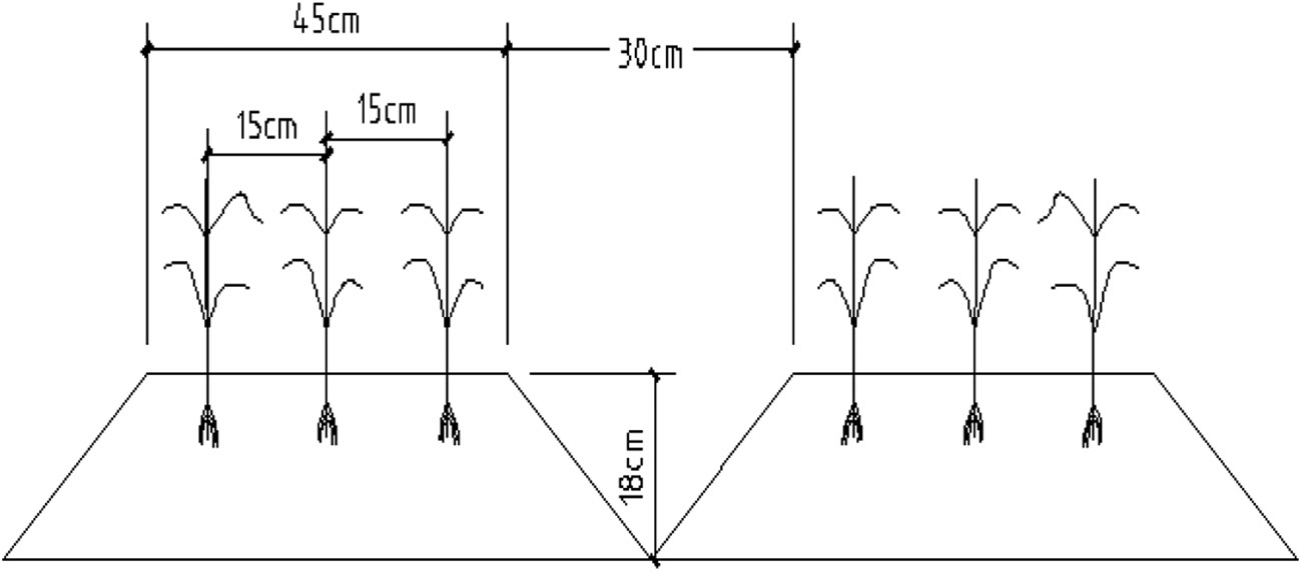
Schematic of ridge culture planting for winter wheat.

**Figure 2:**
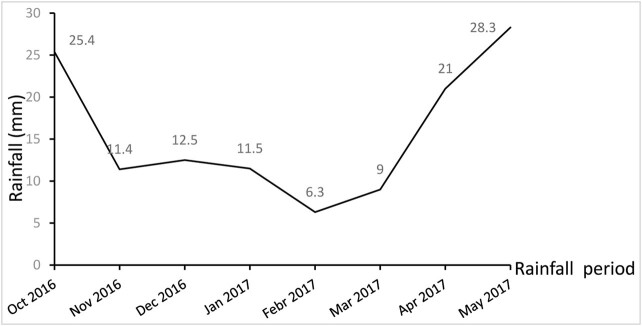
The precipitation distribution during the whole growth stage of winter wheat.

In this experiment, two experimental factors were employed: ditch irrigation regime and lower limit of soil moisture. Two water-saving irrigation regimes, conventional furrow irrigation and alternate furrow irrigation were used. The lower limit of soil moisture was 60, 70, and 80%, with a total of 6 treatments, represented by T1, T2, T3, T4, T5, and T6; each was repeated 3 times. The community area was 20 m × 3 m, with each plot containing four ridges and furrows, developed using a 2BFL-3 multifunctional wheat-ridging planter. Three rows of wheat were planted on the ridge platform, with a ridge width of 45 cm, furrow width of 30 cm, furrow depth of 18 cm, small row spacing of wheat on the ridge was 15 cm, and large row spacing was 45 cm (Figure 1), with ∼3 million plants per hm^−2^. A 1.5 m flat planting isolation area was set between different ridge farming communities to prevent the influence of water infiltration measurement between communities. The former irrigated all the four irrigation ditches in each community; the latter irrigated the first and third ditches in one irrigation period and then irrigated the two non-irrigated ditches for the next irrigation. According to the soil moisture content of the 100 cm-planned wet layer of wheat in different growth periods, irrigation was carried out when the soil moisture content decreased to the lower limit of soil moisture. The amount of irrigation per ditch irrigation was 60 mm, and the amount of irrigation per alternate ditch irrigation was 30 mm. The wheat variety was set as Xinmai 26. From 2016 to 2017, the precipitation in the whole growth period of winter wheat in the test site was 125.4 mm, with the distribution shown in Figure 2.

### 2.3 Experimental methods

#### 2.3.1 Determination of soil water content, farmland water consumption, and water use efficiency

Before wheat sowing and at maturity, the soil samples were taken from the middle of the ridge. The soil moisture content of 0–200 cm soil was measured by a drying method. The soil was taken at 20 cm intervals, a total of 10 layers. Water consumption during the crop growth period was calculated by the water balance formula [31]:

(1) \text{ET}\hspace{.25em}=\hspace{.25em}P+U-R-F\hspace{.25em}+\text{Δ}W\hspace{.25em}+\hspace{.25em}I,]

where Δ*W* is soil water storage consumption; *P* is precipitation in this period (mm); *U* is the amount of groundwater supplied to crops through capillary action (mm); *R* is the surface runoff (mm); *F* is the groundwater supplied (mm); and *I* is the irrigation amount (mm).

The test plot was flat, the groundwater buried depth was less than 5 m, and the precipitation infiltration depth was no more than 2 m, so *U*, *R*, and *F* were all 0.

#### 2.3.2 Crop water productivity

The calculation formula of crop water productivity (CWP) is [42]:

(2) \text{CWP}=Y\text{/ET},]

where *Y* is grain yield (kg hm^−2^), and ET is total water consumption in the whole growth period of crops (m^3^ hm^−2^).

#### 2.3.3 Output and output composition

During the wheat harvest period, the combined harvester in the Wintersteiger community was used to harvest the whole area. During the seed test, spike number, grains per spike, and 1,000-grain weight were measured. After air drying, the grains were weighed and converted into the yield per hectare with 13% water content.

#### 2.3.4 Harvest index

In the mature stage of wheat, a 1 m^2^ sampling area of the wheat field was selected in each plot, all plant samples were obtained, the biological yield was weighed after air drying, the grain quality was threshed and weighed, and the harvest index was calculated as:

(3) \text{Harvest}\hspace{.25em}\text{index}=\text{Grain}\hspace{.25em}\text{yield}/\text{biological}\hspace{.25em}\text{yield}\times 100 \% .]

### 2.4 Data processing and analysis

#### 2.4.1 Use of excel for data sorting and classification

DPS 7.0 software was used for statistical analysis, and a fuzzy comprehensive evaluation model with a two-layer index system was established to evaluate the advantages and disadvantages of different ditch irrigation treatments.

#### 2.4.2 Fuzzy comprehensive evaluation

A fuzzy comprehensive evaluation is an application of fuzzy mathematics, which uses a membership fuzzy matrix to make a comprehensive evaluation of relevant factors. If there are few factors, a system layer can be used. As the selection of furrow irrigation regime is a multi-factor and multi-level evaluation method, it is necessary to use a 2- to 3-layer index system. The description of a fuzzy comprehensive evaluation is as follows:

##### 2.4.2.1 Establishing a multi-level index system

According to different ditch irrigation methods, the formula is *U* = {*u*_1_, *u*_2_, *u*_3_, … *u*_*n*_}. Establishing a primary index system and a secondary index system is necessary.

##### 2.4.2.2 Determining the evaluation factor set

The form of evaluation factor set is: *V* = {*v*_1_, *v*_2_, *v*_3_, … *v*_*m*_}. In this study, three water treatments are used as the evaluation set.

##### 2.4.2.3 Establishing a single factor evaluation matrix:

Each factor *u*_*i*_ (*i* ≤ *n*) can be used to evaluate the results. Since there are different evaluation levels, the evaluation set of each factor can be expressed by the fuzzy vector, *R*_*i*_ = (*r*_*i*1_, *r*_*i*2_, *r*_*i*3_, … *r*_*im*_), *i* = 1, 2, 3, … *n*, *R*_*i*_ ∈ *u*(*V*). All single factor judgments are composed of fuzzy relations. The fuzzy relationship is as follows:

R=\left(\left.\begin{array}{ccccc}{r}_{11} {r}_{12} {r}_{13} \mathrm{..}. {r}_{1m}\\ {r}_{21} {r}_{22} {r}_{23} \mathrm{..}. {r}_{2m}\\ \mathrm{..}. \mathrm{..}. \mathrm{..}. \mathrm{..}. \mathrm{..}.\\ {r}_{n1} {r}_{n2} {r}_{n3} \mathrm{..}. {r}_{nm}\end{array}\right)\right..]

##### 2.4.2.4 Determining index weight

The core of the evaluation system is to calculate the weight that represents the importance of each factor. There are two methods to determine the weight: subjective and objective. Some secondary indicators cannot be determined subjectively, so it is necessary to calculate the weight objectively.

(a) Weight determined by expert prediction
  The importance of each index is compared, and the weight of each index is assigned and calculated based on experts’ knowledge, experience, or preference [45].
(b) Entropy weight method

The entropy weight method is used to obtain the weight of the index objectively. The specific steps are given in the algorithm in ref. [45].

##### 2.4.2.5 Calculating evaluation results

The evaluation results are obtained by membership matrix and weight calculations, and the formula is:

(4) {B}_{i}=\text{WR}({b}_{1},{b}_{2},{b}_{3},\ldots {b}_{m}),]

where *B* is the evaluation result of the index system of all factors.

## 3 Results

### 3.1 Comprehensive evaluation of different furrow irrigation methods based on multi-level fuzzy evaluation

#### 3.1.1 Composition of fuzzy evaluation factor set and its sub-factor set

In this article, the fuzzy comprehensive evaluation of wheat with different furrow irrigation regimes and different water treatments is carried out. The lower limit of 60, 70, and 80% soil moisture of alternate furrow irrigation is expressed by U1, U2, and U3, respectively, and the lower limit of 60, 70, and 80% of soil moisture of furrow irrigation is expressed by U4, U5, and U6, respectively. The furrow irrigation regimes and the lower limit of soil moisture are taken as the primary index. The number of spikes, grains per spike, infertile grains per spike, 1,000-grain weight, yield, harvest index, total water consumption, and water use efficiency are taken as secondary indicators.

#### 3.1.2 Determination of the membership function

The membership function and evaluation matrix are established according to the test data, and the original data in Table 2 are standardized according to the appropriate method in fuzzy mathematics [42] (Table 3).

### 3.2 Secondary evaluation

When evaluating the spike number, grains per spike, 1,000-grain weight, infertile grains number, and harvest index of sub-factors, the entropy weight method is used to determine the weight. The weight of yield, harvest index, irrigation volume, total water consumption, and water use efficiency are determined by expert prediction. After calculation, the weights are 0.067, 0.051, 0.051, 0.035, 0.150, 0.046, 0.250, and 0.350, namely:

{W}_{1}=\{0.067,0.051,0.051,0.035,\hspace{.25em}0.150,\hspace{.25em}0.046,\hspace{.25em}0.250,\hspace{.25em}0.350\}]

{R}_{1}=\left(\begin{array}{cccccccc}0.3189 0.3256 0.2523 0.3141 0.272 0.322 0.3777 0.3327\\ 0.3362 0.3343 0.3892 0.3383 0.3524 0.3814 0.3341 0.3473\\ 0.3449 0.3401 0.3585 0.3476 0.3756 0.2966 0.2883 0.3199\end{array}\right)]

{\tilde{R}}_{1}={W}_{1}\cdot {R}_{1}=(0.3283,0.3468,0.3249)]

{R}_{2}=\left(\begin{array}{cccccccc}0.3178 0.3298 0.2445 0.3244 0.3105 0.3458 0.3591 0.3352\\ 0.3370 0.3375 0.3592 0.3362 0.3429 0.3645 0.3356 0.3464\\ 0.3452 0.3327 0.3963 0.3393 0.3467 0.2897 0.3053 0.3184\end{array}\right)]

{\tilde{R}}_{2}={W}_{1}\cdot {R}_{2}=(0.3315,0.3432,0.3253)]

It can be seen from Table 2 that in the two furrow irrigation regimes, the grain yield of winter wheat increases with the increase in the irrigation amount (*P* < 0.05). However, with sufficient irrigation amounts of T3, T5, and T6, the difference between these three treatments is not significant but is significantly higher than that of other treatments. Since most of the irrigation water is generally maintained in 0–60 cm soil, the surface wetting time is long, and most of the water is used for excess transpiration. The water use efficiency is the lowest under T6 treatment, lower than that of other treatments. In the two furrow irrigation regimes, the water use efficiency increases and decreases with the increasing irrigation. The maximum value of water use efficiency appears at the lower limit of 70% soil moisture, which is significantly higher than other levels for T2. Therefore, a water deficit does not always reduce yield, and winter wheat has a certain ability to adapt to soil drought. This indicates that regulating a moderate water deficit on crops can build a plant group structure with high light efficiency and low water consumption and promote grain development.

Comparing the effects of yield components under different treatments, the number of spike and 1,000-grain weight increases with the increase in soil moisture lower limit under the two furrow irrigation regimes. The difference of 1,000-grain weight under the 70 and 80% soil moisture lower limits is not significant. Due to the limitation of winter wheat storage capacity, 1,000-grain weight will not increase with the increase in irrigation; there is no significant difference in the number of grains per spike among different treatments. With the increase in water demand for reproduction structures in the later growth stages, the water shortage stress of winter wheat will be more severe, which increases the infertile spike number. The harvest index of winter wheat is different among different treatments. When the lower limit of soil moisture is 80%, it is significantly lower than other treatments. More irrigation water promotes the growth and development of winter wheat leaves, roots, and stems, contributing to higher biological yield, a lower harvest index, and lower water use efficiency.

At the same time, it can be seen that the two-factor variance analysis was carried out on each character index of winter wheat according to the two factors of furrow irrigation method and the lower water limit in the experimental design. From the *F* value, it can be concluded that the influence of the lower water limit factor, except for the number of grains per ear, has reached. The number of grains per ear is generally more affected by the characteristics of the variety itself. The effect of furrow irrigation on yield and total water consumption reached a very significant level, and the effect on the number of infertile grains, 1,000-grain weight, and harvest index reached a significant level. The effect of furrow irrigation and water lower limit interaction on grain yield reached a very significant level, and the effect on other traits was not significant.

The secondary evaluation results obtained by fuzzy comprehensive evaluation show that for wheat with alternate furrow irrigation, the T2 evaluation index (0.3468) is the highest, the T1 is the second, and the T3 treatment is the lowest. Under the furrow irrigation treatment, the same evaluation result as the alternative furrow irrigation is obtained, and the T5 evaluation index (0.3432) is higher than T4 and T6. It can be seen from Tables 1–3 that under alternate furrow irrigation and the lower limit of 60% soil moisture, the yield index of wheat is poor with low water stress. But the irrigation regimes cannot be evaluated only by the water use efficiency. For example, with the lower limit of 70% soil moisture, the water use efficiency and harvest index are the highest, and the irrigation effect is the best. With the lower limit of 80% soil moisture, the yield does not increase with increased irrigation, and the water use efficiency is low. Too much water supply leads to high nutrient demand and a low harvest index. Under the condition of conventional furrow irrigation, when the lower limit of soil moisture is 60%, the yield is at the lowest level because of water restrictions. However, compared with the alternate furrow irrigation under the same treatment, the irrigation amount increases by 30 mL, and the grain yield increases significantly because its irrigation uniformity is higher. Under the lower limit of 70% soil moisture, the evaluation index is 0.3432, the irrigation effect is the best, and the coordinated increase in yield, water use efficiency, and the desired harvest index is realized. Under the lower limit of 80% soil moisture, the contribution rate to grain yield and water use efficiency decreases due to excessive irrigation.

**Table 1:** Irrigation schemes for different furrow irrigation regimes

Furrowing irrigation regime	Treatment	Date of irrigation and irrigation quantity/mm									Total irrigation (mm)
		15th Oct 2016	30th Oct 2016	20th Nov 2016	26th Dec 2016	3rd Jun 2017	5th, Apr 2017	19th Apr 2017	6th May 2017	16th May 2017	
Alternate furrow irrigation	T1	30		30	30		30	30			150
	T2	30	30		30		30	30		30	180
	T3	30	30	30	30	30	30	30	30	30	270
Furrow irrigation	T4	60			60			60			180
	T5	60		60			60		60		240
	T6	60		60			60	60		60	300

**Table 2:** Index data of winter wheat yield and water consumption

Irrigation regime	Water treatment	Spike number (104 hm^−^2)	Grain number per spike (grain spike^−1^)	Infertile grain number per spike (grain spike^−1^)	1,000-grain weight (g)	Grain yield (kg hm^−2^)	Economic coefficient	Total water consumption (mm)	Water use efficiency (kg m^−3^)
Alternate furrow irrigation	T1	527.21c	33.6a	5.4a	40.58c	6683.4d	0.38b	367.25d	1.82b
	T2	555.83b	34.5a	3.5bc	43.7ab	7880.72b	0.45a	415.16c	1.9a
	T3	570.15a	35.1a	3.8b	44.9a	8401.25a	0.35bc	481.16a	1.75c
conventional furrow irrigation	T4	528.14c	33.9a	4.7a	43.15b	7496.65c	0.37b	415.49c	1.8b
	T5	560.06b	34.7a	3.2bc	44.72ab	8280.05a	0.39b	444.53b	1.86b
	T6	573.57a	34.2a	2.9c	45.13a	8370.7a	0.31c	488.68a	1.71c
*F* value	Irrigation regimes	3.339	0.074	9.480*	5.786*	51.960**	7.789*	18.339**	3.791
	Water treatment	261.335**	1.225	30.754**	12.955**	213.040**	13.600**	65.338**	31.232**
	Irrigation regimes × water treatment	0.504	0.412	0.627	1.731	21.101**	1.176	3.450	0.220

Note: Different letters after the data indicate significant differences among treatments at the same growth (*p* < 0.05).

**Table 3:** The standardized evaluation matrix of factors affecting winter wheat yield and crop water production

Treatment	Spike number	Grain number per spike	Infertile grain number per spike	1,000-grain weight	Grain yield	Economic coefficient	Total water consumption	Water use efficiency
T1	0.3009	0.3288	0.2632	0.3064	0.2579	0.2966	0.3760	0.2959
T2	0.3459	0.3268	0.3835	0.3445	0.3665	0.3814	0.3278	0.3654
T3	0.3532	0.3444	0.3533	0.3491	0.3756	0.3220	0.2962	0.3387
T4	0.3006	0.3197	0.2107	0.3046	0.2543	0.3137	0.3826	0.2967
T5	0.3438	0.3382	0.3753	0.3469	0.3739	0.3824	0.3239	0.3700
T6	0.3556	0.3421	0.4141	0.3485	0.3718	0.3039	0.2935	0.3333

### 3.3 First level evaluation

Among the primary indicators, because of the small number of indicators and the experience of industry experts in the indicator system, the expert prediction model is adopted to determine the indicator weight, and the following results are obtained:

W=\{0.35,0.35,0.30\},]

R=\left(\begin{array}{cc}0.3283 0.3315\\ 0.3468 0.3432\\ 0.3249 0.3253\end{array}\right).]

\tilde{R}=W\cdot R=(0.3338,\hspace{.25em}0.3337).]

The first-order index evaluation results show that there is little difference between the evaluation index of alternate furrow irrigation (0.3338) and that of conventional furrow irrigation (0.3337). However, the irrigation amount of alternate furrow irrigation is low, and the water use efficiency is high. In the primary index evaluation, under the control of 70% lower limit soil moisture, the harvest index, grain yield, and water use efficiency of the alternate furrow irrigation regime have the best combination. Compared with the test data of T2 (Table 2), the results are consistent with the fuzzy evaluation. The water use efficiency is the highest, and an efficient water-saving irrigation regime is realized. It can be seen from Table 2 that although the water use efficiency of the three soil moisture lower limits of alternate furrow irrigation is higher than that of conventional furrow irrigation, the appropriate furrow irrigation regime should be analyzed and selected according to the water supply conditions in the actual production process, which is not limited to the single factor of water use efficiency.

## 4 Discussion

Crop growth requires appropriate soil moisture. The rainfall characteristics of the experimental site represent the background value of the typical climate characteristics of the region. The total water consumption during the whole growth period of winter wheat accounts for about 20–30%. The contribution rate is general, so supplemental irrigation is carried out in time according to the temporal and spatial requirements of winter wheat growth. Therefore, this study focuses on the impact of irrigation treatment on winter wheat yield. Previous studies have found that moderately regulated deficit irrigation can significantly increase wheat yield and water use efficiency [11,46]. Another research points out that the interval of regulated deficit irrigation also has an impact on crop yield [47]. However, it has also been shown that water deficit can improve water use efficiency in any period but will affect the yield of winter wheat. Winter wheat uses regulated deficit irrigation to reduce irrigation quotas so that crop irrigation systems can be optimized, and crop water stress tolerance and crop yield can be balanced [48]. This study shows that the total water consumption of winter wheat increases with an increase in irrigation, which is consistent with previous research conclusions [49]. However, the increase in the harvest index when the lower limit of soil moisture is 80% is significantly lower than the other two levels. When the lower limit of soil moisture is 70%, the highest harvest index, grain yield, and water use efficiency can be obtained.

The index value of fuzzy comprehensive evaluation also shows that the ditch irrigation regime is best when the lower limit of soil moisture is 70%. It is concluded that a higher yield can be obtained under the alternate furrow irrigation regime because only the ditch on one side of the ridge is irrigated each time. The irrigation amount is reduced, which results in the soil moisture in the middle of the ridge reaching the lower limit of soil moisture faster. Thus, the irrigation times are increased, and the water potential difference between the ridges and ditches on both sides is higher, which is conducive to water infiltration to the ridge. In *Garcinia brasiliensis*, the plants did not reduce stomatal conductance, photosynthesis, photochemical responses, and water use efficiency under moderate water stress (50% and 75%), and there is a remarkable effect of moderate drought on biomass accumulation [50]. In addition, the root signal function generated by the crop side under water stress is used to regulate the stomatal conductance of plant leaves and reduce water loss, to achieve high efficiency. The time interval is short because the alternate furrow irrigation meets the small amount of water demand of the crops each time. In the process of alternate furrow irrigation, the surface soil of adjacent unirrigated ditches remains dry, which increases precipitation storage area, equivalent to the increase in soil water storage capacity, which helps the efficient utilization of precipitation.

## 5 Conclusion

In conclusion, this study improves the calculation method of weights on the basis of fully retaining the advantages of the fuzzy comprehensive evaluation, and a multi-level and multi-factor indices system of furrow irrigation regimes was constructed based on ensuring winter wheat production. Under the two irrigation regimes, conventional furrow irrigation and alternate furrow irrigation, the maximum crop water production of winter wheat of 1.86 and 1.90 kg m^−3^ was obtained under treatment with a 70% lower limit of soil moisture, respectively. Thus, conventional furrow irrigation is recommended in areas with sufficient irrigation water, while alternating furrow irrigation, which is more conducive to reducing the total amount of irrigation required than the conventional furrow irrigation, is suitable for areas with water shortages.
